# Pik3ip1 Modulates Cardiac Hypertrophy by Inhibiting PI3K Pathway

**DOI:** 10.1371/journal.pone.0122251

**Published:** 2015-03-31

**Authors:** Hong Ki Song, Jiyeon Kim, Jong Sub Lee, Kyoung Jin Nho, Hae Chang Jeong, Jihwa Kim, Youngkeun Ahn, Woo Jin Park, Do Han Kim

**Affiliations:** 1 School of Life Sciences and Systems Biology Research Center, Gwangju Institute of Science and Technology (GIST), Gwangju, Republic of Korea; 2 Department of Cardiology, Chonnam National University Hospital, Gwangju, Republic of Korea; Université catholique de Louvain, BELGIUM

## Abstract

Cardiac hypertrophy is an adaptive response to various physiological and pathological stimuli. Phosphoinositide-3 kinase (PI3K) is a highly conserved lipid kinase involved in physiological cardiac hypertrophy (PHH). PI3K interacting protein1 (Pik3ip1) shares homology with the p85 regulatory subunit of PI3K and is known to interact with the p110 catalytic subunit of PI3K, leading to attenuation of PI3K activity in liver and immune cells. However, the role of Pik3ip1 in the heart remains unknown. In the present study, the effects of Pik3ip1 on cardiac hypertrophy were examined. We found that the expression level of Pik3ip1 was markedly higher in cardiomyocytes than in fibroblasts. The interaction of Pik3ip1 with the p110a subunit of PI3K in the heart was identified by immunoprecipitation using neonatal rat cardiomyocytes (NRCM). Approximately 35% knockdown of Pik3ip1 was sufficient to induce myocardial hypertrophy. Pik3ip1 deficiency was shown to lead to activation of PI3K/protein kinase B (AKT)/ mammalian target of rapamycin (mTOR) signaling pathway, increasing protein synthesis and cell size. However, adenovirus-mediated overexpression of Pik3ip1 attenuated PI3K-mediated cardiac hypertrophy. Pik3ip1 was upregulated by PHH due to swimming training, but not by pathological cardiac hypertrophy (PAH) due to pressure-overload, suggesting that Pik3ip1 plays a compensatory negative role for PHH. Collectively, our results elucidate the mechanisms for the roles of Pik3ip1 in PI3K/AKT signaling pathway.

## Introduction

Pathological cardiac hypertrophy (PAH) (i.e. pressure-overload hypertrophy) is an adaptive response to increased workload that initially maintains normal cardiac function. However, prolonged hypertrophic stimuli can lead to fatal heart failure. In contrast, physiological cardiac hypertrophy (PHH) (i.e. exercise training hypertrophy) is the normal response to physical activity characterized by increased thickness of the left ventricular wall and volume. Diverse signaling pathways have been proposed for the different types of hypertrophy [1–3].

PI3K is activated by receptor tyrosine kinases (e.g. insulin and insulin-like growth factor1 (IGF1) receptors). PI3K plays important roles in various signal transduction mechanisms such as cytoskeleton organization, cell growth, and apoptosis [4,5]. The PI3K family can be divided into three major classes according to their amino acid sequences, homology and substrate specificity [6]. Of these, PI3K class Ia and Ib are highly expressed in the heart. Class Ia isoforms are involved in mediating physiological hypertrophy, whereas the class Ib isoform, PI3Kγ, controls myocardial contractility through G protein-coupled receptor signaling [6]. Class Ia PI3Ks are heterodimeric molecules, which include a catalytic 110-kDa subunit (p110α, β, and δ) and a regulatory 85- or 55-kDa subunit (p85/p55). In mammalian cells, the interaction between p110 and p85/p55 is important to achieve PI3K maximal activity [7].

Pik3ip1 is a transmembrane protein that contains an extracellular kringle motif. This protein possesses a domain that is homologous to the PI3K regulatory subunit p85 [8]. Pik3ip1 was originally identified as a binding partner of p110 in the liver and immune cells. It is abundantly expressed in many tissues, including the heart, liver, and lung. Previous studies have revealed that Pik3ip1 acts as a negative regulator of PI3K, playing a key role in the PI3K pathway in the liver and immune cells [9,10]. Because the PI3K pathway is mainly involved in PHH, Pik3ip1 may be a distinct intrinsic regulator of PHH.

The present study demonstrates that Pik3ip1 expressed in cardiomyocytes is involved in the regulation of the PI3K/AKT/mTOR signaling pathways.

## Materials and Method

### Ethics Statement

All animal experiments were approved by the Gwangju Institute of Science and Technology Animal Care and Use Committee. (2014–55)

### Animal models

8 weeks old male (C57BL/6J) mice (body weight 28–33 g) purchased from Samtako Korea were used in all studies.

#### Pathological hypertrophy

Cardiac hypertrophy was induced by TAC operation under anesthesia with intraperitoneal injection of avertin, 2-2-2 tribromoethanol (Sigma) dissolved in tert-amyl alcohol (Sigma). The procedure of operation was followed as previously described [11]. As a control group, sham operation (same procedure except for tying) was done. 1 week or 2 weeks after operation, mice were euthanized by cervical dislocation, and hearts were removed, and then stored in deep freezer at −80°C before protein and RNA extraction.

#### Physiological hypertrophy

For chronic exercise training, mice swam in water tanks for 2 weeks or 4 weeks as described previously [12]. The first day of training consisted of two 10-min sessions separated by at least 4 hrs. The duration of exercise was increased in 10-min increments daily, reaching 90 min, twice daily, by the middle of the second week. This duration of exercise was maintained until 2 weeks or 4 weeks. Trained mice were euthanized 24 h after the last training session to exclude any acute effect of exercise by cervical dislocation. Dissected hearts were frozen and then stored in deep freezer at -80°C before protein RNA extraction.

### Antibodies and chemicals

The anti-α-actinin antibody (A7811) was from Sigma-Aldrich. The anti-phospho AKT (#9271), anti-AKT (#9272), anti-phospho mTOR (#2971), anti-mTOR (#2972), anti-phospho p70s6k (#9206), anti-p70s6k (#9202), anti-phospho eEF2 (#2331), anti-eEF2 (#2332), anti—phospho ERK1/2 (#9101), anti-ERK1/2 (#9102), anti-Vimentin (#5741) and anti-p110α (#4249) antibodies were from Cell Signaling Technology. The anti- α-Tubulin (sc-5286), anti-p110α (sc-7174) and anti P110γ (sc-7177) antibody were from Santa Cruz Biotechnology. The anti-Pik3ip1 (16826-1-AP) antibody was from proteintech. AngII (Angiotensin II) was purchased from Sigma-Aldrich, LY294002 was from Calbiochem and Human IGF-1 (insulin-like growth factor-1) was from R&D Systems.

### Isolation of rat neonatal cardiomyocytes

Neonatal cardiomyocytes (NRCMs) from 1-3-day-old Sprague-Dawley rat pubs were euthanized by CO^2^ followed by decapitation were isolated using the neonatal cardiomyocyte isolation system (Worthington Biochemical) as described previously [13]. Briefly, rat neonatal hearts were minced with sterile scissors and dissociated to release ventricular cardiomyocytes by treatment with first using a trypsin solution (50 *μ*g/ml) for 18 h at 4°C, and then with a collagenase solution (300 units/ml) at 37°C for 40 min. After non-myocytes were removed by pre-plating on untreated plastic culture dishes for 1 h, isolated cardiomyocytes were cultured in DMEM (Dulbecco’s modified Eagle’s medium)/M199 medium supplemented with 1 mM glutamine, 10% FBS, 0.1 mM bromodeoxyuridine, 100 units/ml penicillin and 100 *μ*g/ml streptomycin.

### Silencing of mRNA and protein expression by siRNA transfection

The primary neonatal cardiomyocytes were serum-starved for 24 h and then transfected with 50 nM siRNA for Pik3ip1 and negative control (Bioneer) by using DharmaFECT-1 reagent according to the manufacturer’s instructions. The sense sequences of siRNAs used were: Negative Control siRNA (siNegiative), 5’-GUGCGUUGCUAGUACCAACdUdU-3’; rat Pik3ip1 siRNA (siPik3ip1), 5’-GCG UUACUAUGACGGUAAUUAdUdU- 3’. mRNA and protein expression were measured 48 h after transfection by qRT-PCR and Western blotting, respectively.

### Generation of Recombinant Adenovirus

Replication‐deficient adenoviruses for hrGFP and HA tagged mouse Pik3ip1 were generated using the AdEasy adenoviral system (Agilent) according to the manufacturer’s instructions. NRCMs were infected with adenovirus at a multiplicity of infection of 20 plaque‐forming units, and cultured for 24 h before experimentation.

### Protein synthesis Assay

The rate of protein synthesis was determined by the incorporation of ^3^H-Leucine (Amersham Biosciences) as described previously [14]. Briefly, NRCMs were plated in 24-well plates. ^3^H-Leucine (1 *μ*Ci/ml) was used to label newly translated proteins for 6 h. The cells were treated with 5% trichloroacetic acid at 4°C for 30 min and then lysed with NaOH (0.1 N) at 37°C for 30 min. Radio activity was measured using a liquid scintillation counter.

### Cell surface area measurement

After cardiomyocytes were grown in serum-starved conditions on 18 mm cover slips, siRNA were transfected into cells with DharmaFECT-1 reagent. Twenty four hours after transfection, cells were incubated for another 24 h with new media, and then fixed with 4% paraformaldehyde for 15 min. The cells were permeabilized with 0.1% Triton X-100, followed by blocking with 5% BSA. For staining of cardiomyocytes, cells were sequentially incubated with anti-α-actinin antibody, followed by anti-mouse secondary antibody conjugated to Alexa Fluor 594 (Invitrogen). Nuclear staining was performed with DAPI (Molecular Probes). The prepared cells were examined using an IX81 inverted microscope equipped with an FV1000 spectral confocal apparatus (Olympus) and analyzed using Metamorph software (Universal Imaging Corporation).

### Quantitative real-time PCR (qRT-PCR) and reverse-transcriptional PCR (RT-PCR)

After extraction of total RNA from rat neonatal cardiomyocytes using TRIzol reagent (Invitrogen), 400 ng of RNA was reversetranscribed into cDNA using the Prime Script RT reagent kit (TaKaRa) according to the manufacturer’s instruction. qRT-PCR assays were followed as previously described [15]. Briefly, qRT-PCR assays were performed using TOPreal qPCR PreMix (Enzynomics) under the following two-step conditions: denaturation at 95°C for 5 seconds; and annealing and extension at 60°C for 40 seconds, for a total of 40 cycles. The 18S transcript was used as an endogenous reference to assess the relative level of mRNA transcript. RT-PCR assays were performed on a TAKARA thermal cycler TP600 (TaKaRa) using nTaq-HOT DNA polymerase (Enzynomics) under the following 3 step conditions: denaturation at 94°C for 30s, annealing at 55–60°C for 30s and extension at 72°C for 40s with total 30–35 cycles. The sequences of specific primers for 18S and each of the transcripts are shown in Table A in S1 File.

### Co-immunoprecipitation assays

Mouse hearts or NRCM were solubilized in 1% Triton X-100 Lysis buffer containing 25 mM Tris/HCl, pH 7.4, 150 mM NaCl, 0.5mM EDTA, 1% Triton X-100 and protease inhibitor cocktail (Calbiochem) for 30 min at 4°C. After centrifugation at 13000 g for 30 min at 4°C to remove unsolubilized vesicles and precipitates, the supernatant was further cleared by incubation with Dyna protein A magnetic beads for 30 min 4°C. The cleared supernatants were then incubated with antibody overnight at 4°C. Protein A-Dyna magnetic Beads (Invtrogen) (for the rabbit primary antibody) were then used to precipitate the antibodies followed by three washes with 1% Triton X-100 Lysis buffer.

### Measurement of PI3K (p110α) activity

p110α activity were measured by PI3-Kinase activity ELISA Kit (Echelon) according the manufacture protocol with some modifications. After treatment, NRCMs were lysed with ice-cold buffer containing 137 mM NaCl, 20 mM Tris pH7.4, 1 mM Cacl2, 1 mM Mgcl2, 1% Nonidet P-40, phosphatase inhibitor cocktail (Roche) and protease inhibitor cocktail (Roche) and solubilized by continuous stirring for 30 min at 4°C. After centrifugation (16,000 g, 15 min), the supernatant was collected, and 800 mg of homogenate (in 600 *μ*l) was immunoprecipitated with p110α antibody from santacruz (H-201). After 4hr incubation at 4°C, Protein A-Dyna magnetic Beads (Invtrogen) was added, and the immune complex was washed four times with buffer (100 mM NaCl, 1 mM Na3VO4, and 20 mM HEPES, pH 7.5) and resuspended in 40 *μ*l of KBZ buffer (180 mM NaCl-20 mM HEPES, pH 7.5). The kinase reaction was run for 3 hr at room temperature. Converted PIP3 level from PIP2 was measured by Virtor3 at 450 nM.

### Western blot assay

Cardiomyocytes were solubilized in lysis buffer containing 10 mM Tris/HCl (pH 7.4), 1% SDS, phosphatase inhibitor cocktail (Roche) and protease inhibitor cocktail (Roche). After centrifugation at 12000 rpm for 10 min at 4°C, the supernatant was analyzed for protein concentration using the BCA method, and then denatured at 95°C for 5 min with SDS sample buffer containing DTT. Proteins (10 ~ 40*μ*g) were separated by SDS/PAGE (8–15% gel) and subsequently transferred on to PVDF membranes. After blocking with 5% skim milk, the proteins were incubated with primary antibodies at 4°C overnight. Horseradish peroxidase-conjugated secondary antibodies were then applied at room temperature for 1 h, and specific protein bands were detected using ECL (Amersham).

### Isolation of adult mouse ventricular myocytes

Ventricular myocytes were isolated from mouse hearts using the method as described previously [11]. Male mice of 8–12 weeks of age (25–32 g) were used for the study. In brief, afterheparin (50 units) injection, animals were sacrificed by cervical dislocation. The heart was quickly removed from the chest and the aorta was retrogradely perfused at 37°C for 3 min with calcium-free Tyrode buffer (137 mM NaCl, 5.4 mM KCl, 1 mM MgCl2, 10 mM glucose, 10 mM HEPES, 10 mM 2, 3-butanedione monoxime (BDM; Sigma), and 5 mM taurine (Sigma) (pH 7.4)) gassed with 95% O2/5% CO2. The enzymatic digestion was initiated by adding collagenase type B (0.35 U/ml; Roche) and hyaluronidase (0.1 mg/ml; Sigma) to the perfusion solution. When the heart became swollen after 10 min of digestion, the left ventricle was quickly removed, cut into several chunks, and further digested in a shaker (60–70 rpm) for 10 min at 37°C in the same enzyme solution. The supernatant containing the dispersed myocytes was filtered through a cell strainer (100 μm, BD Falcon) and gently centrifuged at 500 rpm for 1 min. Extracellular Ca^2+^ was incrementally added back to 1.25 mM over a span of 30 min to avoid Ca^2+^ paradox. This procedure usually yielded more than 50% viable rod-shaped ventricular myocytes with clear sarcomere striations. Myocytes with obvious sarcolemmal blebs or spontaneous contraction were not used.

### Echocardiography

Echocardiography was performed to measure LV function. Mouse were anesthetized with 50 mg/kg ketamine hydrochloride and 10 mg/kg xylazine hydrochloride for echocardiographic examination. Echocardiographic studies were performed with a 15-MHz linear array transducer system (iE33 system; Philips Medical Systems, Andover, MA, USA) by an expert who was not aware of experimental conditions to exclude bias. FS (%) = [(LVIDd − LVIDs) / (LVIDd)] × 100, where LVIDd is LV internal dimension on end diastole and LVIDs is LV internal dimension on end systole. All measurements were averaged for three consecutive cardiac cycles.

### Statistics

Data were collected from at least three independent experiments carried out using independent cultures and then quantified. The statistical significance of differences between means was assessed using unpaired Student’s t tests or ANOVA. Data are presented as means ± S.E.M. Statistical significance was considered at P < 0.05 or <0.01.

## Results

### Pik3ip1 is expressed in neonatal rat cardiomyocytes and interacts with p110α

**Fig 1:**
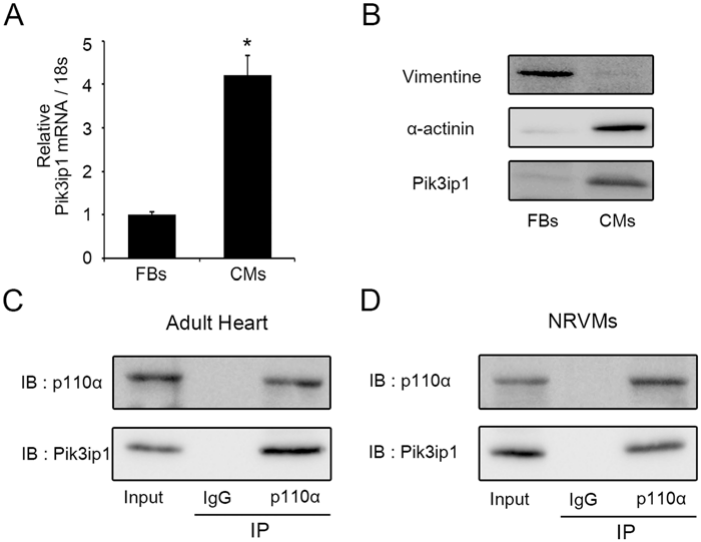
Pik3ip1 is enriched in neonatal rat ventricular cardiomyocytes and interacts with p110α. (**A**) mRNA levels of Pik3ip1 were measured in cardiomyocytes (CMs) and fibroblasts (FBs) using quantitative reverse transcription PCR (qRT-PCR) (n = 3, * p < 0.05, t test). (**B**) Western blot analysis was performed to compare CMs and FBs using anti-Pik3ip1, Vimentin, and α-actinin antibodies. (**C**, **D**) The interaction between Pik3ip1 and p110α was analyzed in adult mouse heart tissue (**C**) and NRCMs (**D**) using anti-p110α or anti-Pik3ip1 antibodies.

Because Pik3ip is known to be expressed in mouse heart tissue [8], Pik3ip expression was examined in two cardiac cell types, cardiomyocytes and fibroblasts, using neonatal rat heart. The results showed that Pik3ip1 was enriched in cardiomyocytes, but not in fibroblasts, at both the mRNA and protein levels (Fig 1A and 1B). The validities of the two cardiac fractions were confirmed by examining the expression levels of α-actinin (*Actn1*), a myocyte-specific protein [16], and vimentin (*Vim*), a fibroblast-specific protein [17]. Fig. A in S1 File show that *Actn1* and *Vim* were significantly enriched in the cardiomyocyte and fibroblast fractions, respectively.

Next, we performed immunoprecipitation (IP) to determine whether Pik3ip1 interacts with p110α or p110γ (catalytic subunit) of PI3K in the heart. Western blot analysis showed that IP samples precipitated with p110α antibody contained Pik3ip1 (Fig 1C and 1D). However, IP samples precipitated with p110γ antibody had no band for Pik3ip1 (Fig B in S1 File), suggesting that Pik3ip1 interacts with p110α protein in the heart.

### Downregulation of Pik3ip1 by siRNA in NRCM-induced myocyte hypertrophy

**Fig 2:**
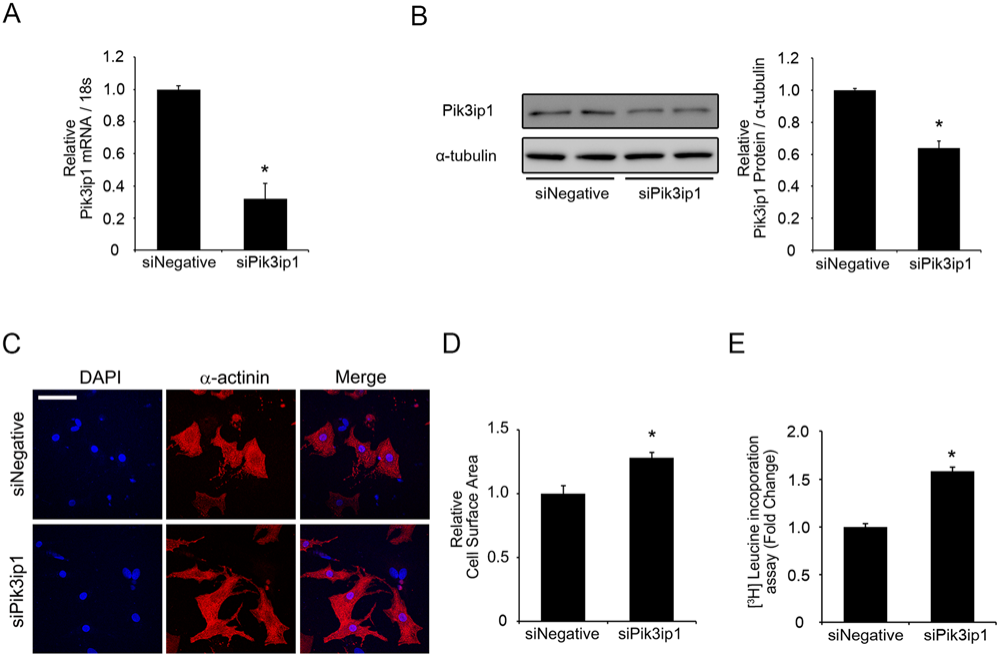
Silencing of Pik3ip1 induces cardiomyocyte hypertrophy. NRCMs were transfected with the indicated siRNA for 24 h and subsequently serum-starved for 24 h. (**A**) qRT-PCR analysis of the mRNA transcripts for Pik3ip1 in 50 nM siNegative or 50 nM siPik3ip1-transfected NRCMs. mRNA expression was normalized to 18S. (n = 3, ** p < 0.01, t test). (**B**) Representative immunoblot images (left) and quantified graphs (right) of Pik3ip1 protein in siNegative or siPik3ip1-transfected NRCMs. *α*-Tubulin served as an internal control. (n = 3, * p < 0.05, t test). (**C**) Representative images of NRCMs stained with anti-α-actinin antibody and DAPI in siNegative or siPik3ip1-transfected NRCMs. Scale bar: 100 μm. (**D**) Quantification of the relative cell surface areas. (n = 5, ** p < 0.01, t test). (**E**) Effect of Pik3ip1 knockdown on protein synthesis. Leucine incorporation was determined as described in the **Materials and Methods**. (n = 12, ** p < 0.01, t test).

Given that the PI3K pathway is essential for triggering PHH, and that Pik3ip1 is known to be a negative regulator of PI3K [8,9], the possibility that Pik3ip1 is an intrinsic inhibitor of physiological hypertrophy was examined by siRNA-mediated Pik3ip1 downregulation using NRCM. NRCMs were transfected with 50 nM negative control siRNA (siNegative) or 50 nM siRNA for Pik3ip1 (siPik3ip1). As shown in Fig 2A, the expression of Pik3ip1 mRNA in siPik3ip1-transfected NRCMs was significantly downregulated compared with the negative control (siNegative). A western blot assay also showed downregulation of Pik3ip1 at the protein level (≒35%) (Fig 2B). Under the same experimental conditions, cell size and protein synthesis were examined for each experiment. Pik3ip1 siRNA-transfected cells showed significantly increased cell size, compared with the negative siRNA-transfected NRCMs (Fig 2C and 2D). To further verify the development of hypertrophy in Pik3ip1-deficient NRCMs, protein synthesis was examined using a ^3^H-Leucine incorporation assay. ^3^H-Leucine uptake was significantly higher in Pik3ip1-deficient NRCMs than in negative controls (Fig 2E).

Because it is known that PAH is accompanied with increasing the expression of the fetal genes, such as *Nppa* (natriuretic peptide A; also known as ANF (atrial natriuretic factor)), *Nppb* (natriuretic peptide B; also known as BNP (brain natriuretic factor)), and *Myh7* (myosin heavy chain; also known as β-MHC) [15,18], we measured the expression of fetal genes, using qRT-PCR. Although Pik3ip1-deficiency increased cell size and protein synthesis, the expression of the fetal genes *Nppa*, *Nppb*, *and Myh7* did not change (Fig. C in S1 File), suggesting that Pik3ip1 deficiency does not induce pathological hypertrophy.

### Knockdown of Pik3ip1 stimulated PI3K, AKT, and downstream molecules controlling protein synthesis and cell growth

**Fig 3:**
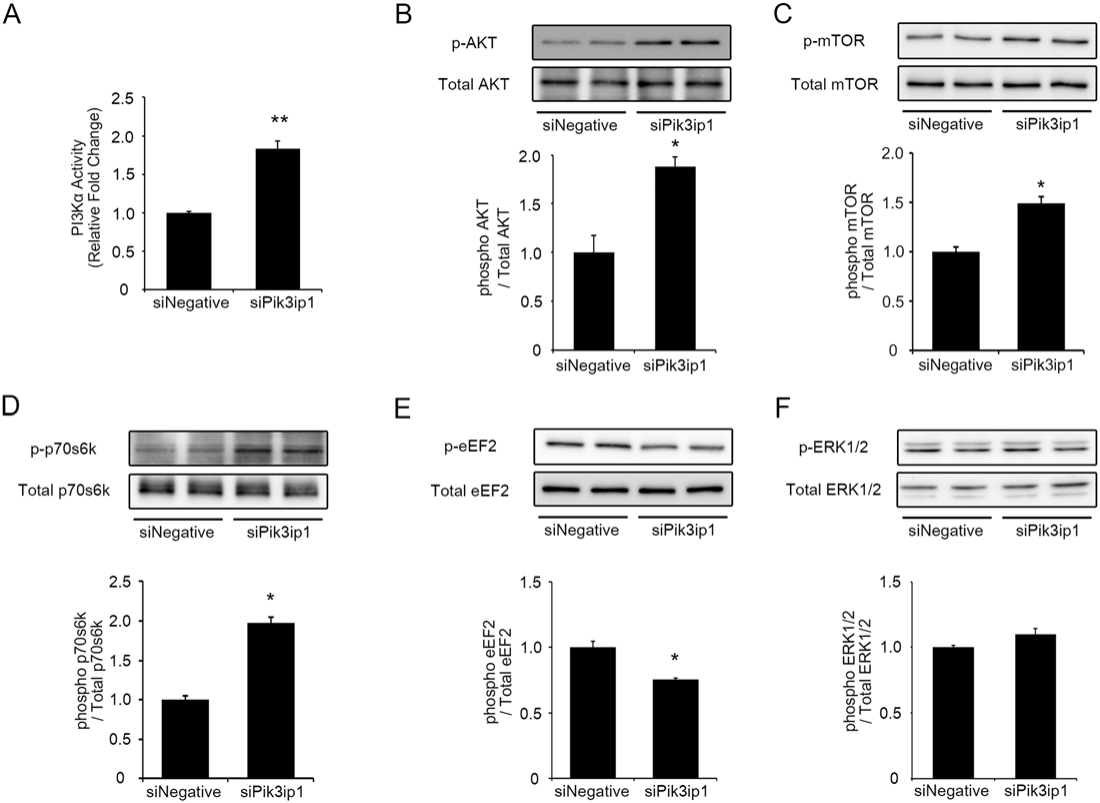
The effects of silencing Pik3ip1 on the PI3K/AKT/mTOR pathway. NRCMs were transfected with the indicated siRNA for 24 h and subsequently serum-starved for 24 h. **(A)** PI3K activity (p110α) in the siNegative and siPik3ip1-transfected NRCMs. Western blot analyses of samples from siNegative and siPik3ip1-transfected NRCMs were performed after 48 h. (**B–E**) Representative immunoblot images of phosphorylated protein and total protein (upper), and the ratio of phosphorylated protein/total protein (bottom) for (**B**) AKT, (**C**) mTOR, (**D**) p70s6k, and (**E**) eEF2. **(F)** ERK 1/2 (n = 3, * p < 0.05 and ** p < 0.01, t test)

To test whether the increased hypertrophy and protein synthesis observed in Pik3ip1-deficient NRCMs was due to PI3K activation, we measured PI3K (p110α) activity in siNegative or siPik3ip1 transfected NRCMs. After P110α immunoprecipitation using a specific antibody, we conducted a PI3-kinase assay with phosphatidylinositol 4,5-bisphosphate (PIP_2_), *in vitro*, and measured the converted phosphatidylinositol (3,4,5)-trisphosphate (PIP_3_) levels by enzyme-linked immunosorbent assay (ELISA). As shown in Fig 3A, Pik3ip1 knockdown increased p110α activity. Next, we measured AKT phosphorylation, the downstream substrate of PI3K. As shown in Fig 3B, Pik3ip1 knockdown markedly increased AKT phosphorylation, suggesting that Pik3ip1 inhibits PI3K kinase activity. Tests were conducted to further assess the functional consequences of Pik3ip1 knockdown on downstream signaling molecules, phosphorylation of mTOR and p70s6k, downstream molecules in the PI3K pathway that are known to control protein translation and cardiomyocyte growth [19,20]. We found that Pik3ip1 knockdown significantly increased mTOR and p70s6k phosphorylation, indicative of their activation (Fig 3C and 3D). Another important molecule downstream of AKT that controls protein synthesis is the elongation factor eEF2, which is known to be activated by dephosphorylation [21]. Using a phospho-eEF2 specific antibody, we found that its phosphorylation level was significantly lower in Pik3ip1-deficient NRCMs (Fig 3E). Furthermore, we examined the phosphorylation level of a pro-hypertrophy protein, extracellular signal-regulated kinase ½ (ERK1/2) [22]. As shown in Fig 3F, ERK1/2 phosphorylation level was not changed. Collectively, the results support the hypothesis that Pik3ip1 deficiency increased protein synthesis as well as cell size by modulating mTOR, p70s6k, and eEF2, the targets of AKT.

### Pik3ip1-deficiency-induced protein synthesis and hypertrophy is due to activation of PI3K

**Fig 4:**
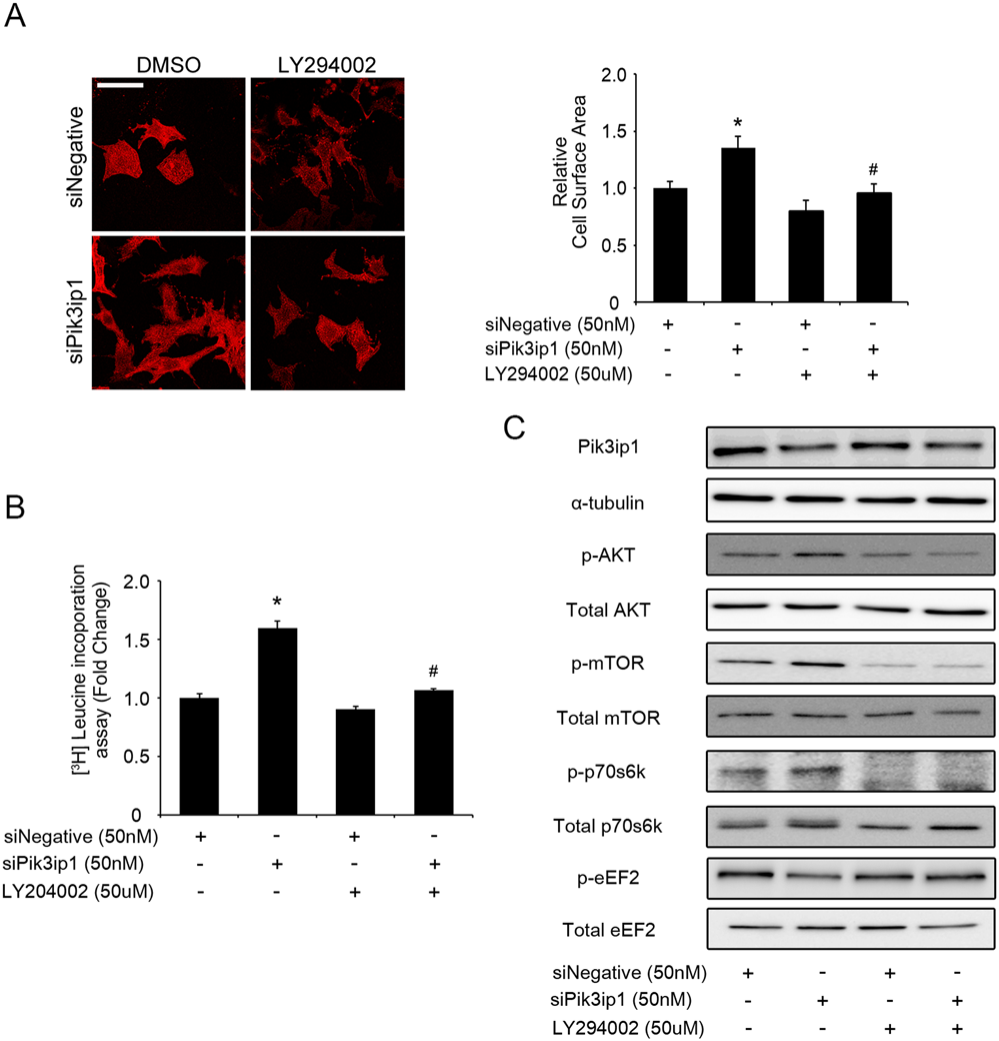
Pik3ip1 silencing-induced cardiomyocyte hypertrophy is dependent on PI3K activity. NRCMs were transfected with siNegative and siPik3ip1 for 24 h and subsequently treated with 0.05% DMSO or LY294002 for 24 h. (**A**) Representative images (left) of NRCMs stained with anti-α-actinin antibody in siNegative or siPik3ip1-transfected NRCMs with or without LY294002. Scale bar: 100 μm. Quantification of relative cell surface areas (right). (n = 5, * p < 0.05 compared with siNegative-transfected NRCMs treated with DMSO, and ## p < 0.01 compared with siPik3ip1-transfected NRCMs treated with DMSO, ANOVA). (**B**) All siRNA-transfected NRCMs were incubated with DMSO or LY294002, after which protein synthesis was assessed using a leucine incorporation assay. (n = 12, ** p < 0.01 compared with siNegative-transfected NRCMs treated with DMSO and ## p < 0.01 compared with siPik3ip1-transfected NRCMs treated with DMSO, ANOVA). (**C**) The signaling molecules involved in the PI3K pathway in the 4 different types of samples (siNegative, siPik3ip1, siNegative plus LY294002, and siPik3ip1 plus LY294002) were verified by the indicated antibodies.

To test whether PI3K inactivation restores Pik3ip1-deficient NRCM phenotypes, we performed a study with LY294002, a specific inhibitor of PI3K, using Pik3ip1-deficient NRCMs [23]. First, we investigated the effect of LY294002 on cell size after transfection of each siRNA in NRCMs. Twenty-four hours after siRNA transfection, the medium, which included 0.05% DMSO or 10 μM LY294002, was changed. As shown in Fig 4A, inhibition of PI3K activity by LY294002 attenuated the Pik3ip1-deficiency-mediated increase in cell size in NRCMs. In parallel, we measured protein synthesis of ^3^H-leucine using siPik3ip1-transfected NRCMs. As expected, protein synthesis was significantly decreased in LY294002-treated NRCMs, compared to cells treated with DMSO only (Fig 4B). We then examined the activation state of the PI3K/AKT/mTOR pathway by measuring the phosphorylation levels of each protein in siPik3ip1-transfected NRCMs. Phosphorylated forms of AKT, mTOR, and p70s6k were decreased in LY294002-treated NRCMs, compared to DMSO-treated cells. In contrast, phosphorylated eEF2, which was dephosphorylated by siPik3ip1, was increased by LY294002 treatment (Fig 4C). Collectively, the results suggest that Pik3ip1 deficiency activates the PI3K pathway and induces cardiac hypertrophy.

### Overexpression of Pik3ip1 attenuates IGF1-induced cardiac hypertrophy

**Fig 5:**
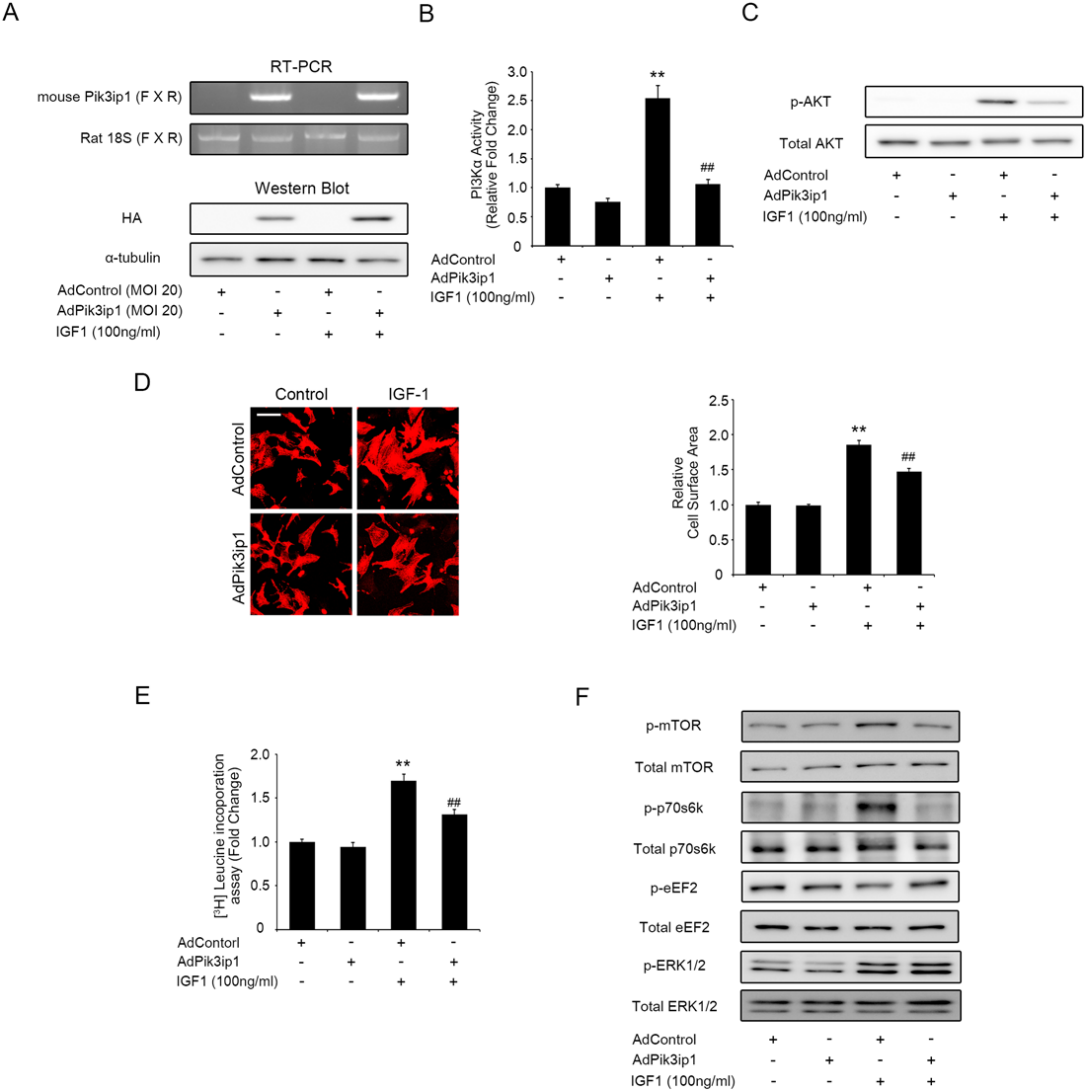
Pik3ip1 attenuates IGF1-induced cardiomyocyte hypertrophy. NRCMs were infected with the indicated adenovirus for 24 h and subsequently treated with or without 100 ng/ml IGF1. (**A**) Exogenous HA-tagged mouse Pik3ip1 expression was verified by RT-PCR and western blot analyses. Exogenous Pik3ip1 mRNAs were amplified using a mouse Pik3ip1-specific primer set (Table A in S1 File). The corresponding cell lysates from adenovirus-infected NRCMs were analyzed by immunoblot assay with anti-HA and anti-α-tubulin antibodies. (**B-C**) Adenovirus-infected NRCMs were incubated for 30 min with or without IGF1, after which p110α activity **(B)** and AKT phosphorylation **(C)** was assessed. (**D**) Representative images (left) of NRCMs stained with anti-α-actinin antibody in AdControl or AdPik3ip1-infected NRCMs for 24 h with or without IGF1. Scale bar: 100 μm. Quantification of relative cell surface area (right). (n = 5, ** p < 0.01 compared with AdControl-infected NRCMs and ## p < 0.01 compared with AdControl-infected NRCMs treated with IGF1, ANOVA). (**E**) Adenovirus-infected NRCMs were incubated for 24 h with or without IGF1, after which protein synthesis was assessed using a leucine incorporation assay (n = 5, ** p < 0.01 compared with AdControl-infected NRCMs and ## p < 0.01 compared with AdControl-infected NRCMs treated with IGF1, ANOVA). **(F)** Extracts from adenovirus-infected NRCMs treated for 30 min with or without IGF1 were verified by the indicated antibodies.

The finding that Pik3ip1 deficiency induces cardiac hypertrophy through PI3K/AKT activation led us to examine whether Pik3ip1 overexpression has the opposite effect. Thus, we further examined whether Pik3ip1 overexpression inhibits the PI3K pathway. A recombinant adenovirus system expressing HA-tagged mouse Pik3ip1 (AdPik3ip1) was generated for the study. A hrGFP-expressing adenovirus system served as a control (AdControl). RT-PCR and western blot analyses revealed a specific band for Pik3ip1 in NRCMs infected with AdPik3ip1 (MOI 20), but not in NRCMs infected with AdControl (MOI 20) (Fig 5A). Next, the effect of Pik3ip1 overexpression on PI3K activity (p110α) in IGF1-treated NRCMs was examined. Twenty-four hours after virus infection, the cells were further stimulated by IGF1 for 30 min. As shown in Fig 5B, increased p110α activity by IGF1 treatment was significantly attenuated by Pik3ip1 overexpression. Next, we measured AKT phosphorylation. AKT phosphorylation by IGF1 treatment was significantly decreased in Pik3ip1-overexpressing NRCMs (Fig 5C). Given that Pik3ip1 overexpression inhibits IGF1-induced p110α activity and AKT activation, we hypothesized that Pik3ip1 overexpression could attenuate NRCM hypertrophic growth. As predicted, IGF1 treatment for 24 h significantly increased protein synthesis and cell size in AdControl-infected NRCMs, whereas the IGF1-induced increase in protein synthesis and cell size was significantly suppressed in AdPik3ip1-infected NRCMs (Fig 5D and 5E). Furthermore, phosphorylation of signaling proteins such as mTOR, p70s6k, and eEF2 in the PI3K/AKT pathway was examined. As shown in Fig 5F, AdPik3ip1-infected NRCMs showed attenuated phosphorylation of mTOR and p70s6k after IGF1 treatment. In parallel, eEF2 phosphorylation was higher in AdPik3ip1-infected NRCMs after IGF1 treatment. However, IGF1 induced ERK1/2 phosphorylation was not attenuated by Pik3ip1 overexpression (Fig 5F). Next, we examined the effect of Pik3ip1 overexpression in angiotensin II (AngII) treated samples. Although overexpression of Pik3ip1 partly attenuated AKT phosphorylation induced by AngII treatment, protein synthesis was not affected (Fig D in S1 File). Collectively, the results suggest that Pik3ip1 inhibits the activation of the PI3K pathway and attenuates IGF1-induced hypertrophic growth in NRCMs.

### Exercise-training animal model shows that Pik3ip1 expression is upregulated by 4-weeks exercise training

Our findings that Pik3ip1 expression levels modulate cardiomyocyte hypertrophy through the PI3K pathway, led us to test Pik3ip1 expression levels in mouse models of cardiac hypertrophy. To generate PHH and PAH mouse models, C57BL/6J mice were subjected to swim training for 2 and 4 weeks and transverse aortic constriction (TAC) for 1 and 2 weeks. We examined the heart weight/body weight ratio of these mice. One-week, 2-weeks TAC, and 4-weeks exercised mice presented a significantly increased heart weight/body weight ratio, but 2-weeks exercised mice did not display any change (Table B in S1 File). Next, we used two-dimensional echocardiography to examine the heart function. Mice with 1-week and 2-weeks TAC operation showed an enlarged left ventricular internal dimension (LVID) and post-wall thickness (LVPW) at the diastole compared to the sham operated mice. Left ventricular fractional shortening (FS) was significantly decreased in 1-week and 2-weeks TAC operated mice. However, 2-weeks and 4-weeks exercise training did not alter FS (Table B in S1 File). To further verify PHH and PAH models, we measured the expression level of fetal genes by qRT-PCR. As shown in Fig. E in S1 File, *Nppa*, *Nppb*, and *Myh7* were significantly upregulated in the TAC model, but were not significantly increased in the exercised model.

**Fig 6:**
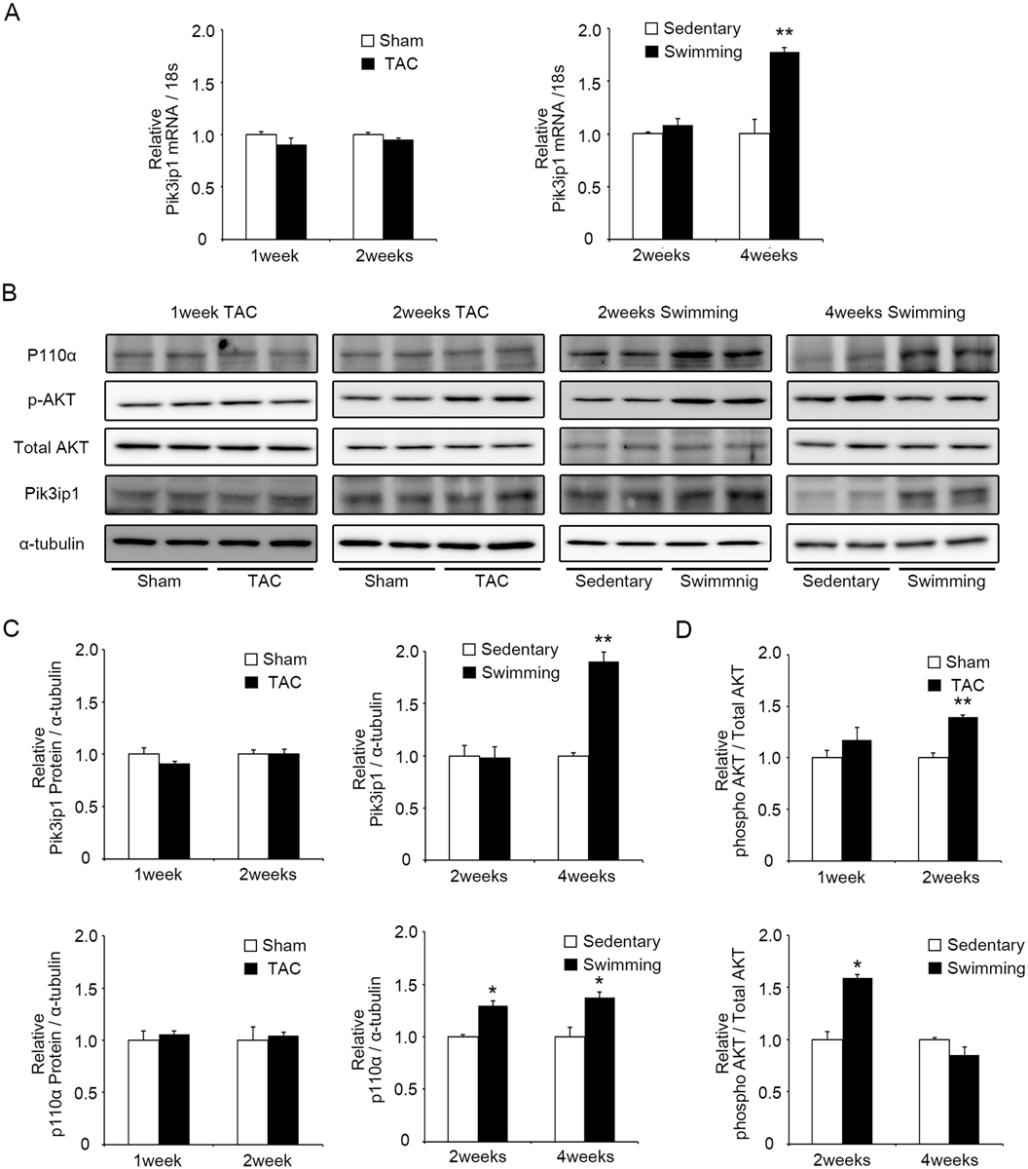
Pik3ip1 expression was upregulated in 4-weeks exercise-induced hypertrophic hearts. **(A)** qRT-PCR analysis of transcripts for *Pik3ip1* in 1-week TAC, 2-weeks TAC, 2-weeks exercised and 4-weeks exercised mice hearts. mRNA expression was normalized to 18S. (n = 3, ** p < 0.01 compared with compared with the Sham or Sedentary mice, t test). **(B)** Representative immunoblot images p110α, AKT, Pik3ip1 and α-tubulin protein expression in 1-week TAC, 2-weeks TAC, 2-weeks exercised and 4-weeks exercised mice hearts. **(C)** Quantification of Pik3ip1 and p110α protein expression in 1-week TAC, 2-weeks TAC, 2-weeks exercised and 4-weeks exercised mice hearts.α-tubulin served as an internal control. (n = 3, * p < 0.05 compared with the Sham or Sedentary mice, t test) **(D)** Quantification of phosphorylated AKT in 1-week TAC, 2-weeks TAC, 2-weeks exercised and 4-weeks exercised mice hearts. Total AKT served as a control. (n = 3, * p < 0.05 compared with the Sham or Sedentary mice, t test).

We examine Pik3ip1expression level in these animal models. The results showed that Pik3ip1 was significantly upregulated in the 4-weeks exercised model both at the mRNA and protein levels (Fig 6A–6C). However, no significant change in Pik3ip1 expression was observed in the 2-weeks exercise, 1-week, and 2-weeks TAC animals (Fig 6A–6C). Because Pik3ip1 is a negative regulator of PI3K, we measured p110α expression and AKT phosphorylation. As shown in Fig 6B and 6C, p110α expression increased in both exercise models. Two-weeks TAC and exercised mice presented an increase in AKT phosphorylation. However, AKT phosphorylation was attenuated in the heart of 4-weeks exercised mice although p110α was increased. We next examined Pik3ip1expression level in myocytes and fibroblasts in the heart of 2-weeks TAC and 4-weeks exercised mice. In 2-weeks TAC operated mouse hearts, Pik3ip1 expression was not changed in both the cell types. However, Pik3ip1 expression was upregulated in myocytes, but not in the fibroblasts in the heart of 4-weeks exercised mice (Fig F in S1 File). These data suggest that the increased Pik3ip1 in myocytes from 4-weeks exercised mice could contribute to the attenuation of AKT phosphorylation.

## Discussion

Cardiac hypertrophy is a central feature of the pathological and physiological remodeling of the heart. PAH induced by pathological stimuli, such as pressure overload, compensates for the increased workload, but its progression generally leads to heart dysfunction and failure [1,24]. In contrast, PHH induced by physiological stimuli such as exercise training is characterized by increased left ventricular volume and a proportional increase in wall thickness and mass [25]. Both types of cardiac hypertrophy are complex processes involving the coordinated action of various signaling pathways [19,24,26]. A number of studies have suggested that hypertrophic responses are associated with increased cardiomyocyte size and increased protein synthesis owing to involvement of key signaling molecules such as PI3K, PKC, and MAPKs. These signaling pathways are known to directly modulate hypertrophic growth by altering nuclear gene expression and by increasing the rates of cytoplasmic protein translation [4,27,28].

PI3K is known to play a central role in regulation of proliferation, cell growth, and cell survival [19,29]. In particular, PI3K has been shown to regulate the size of organs and cells. Of several isoforms, PI3Kα, which consists of a heterodimer of a p110 catalytic subunit and a p85 regulatory subunit [30], is activated by insulin or IGF1, and has been proposed to regulate PHH [12]. Knockout or knockdown of p110α has been shown to reduce PI3K activity and to reduce heart size, due to diminished cardiomyocyte size [31]. Conversely, overexpression of p110α has been shown to lead to heart hypertrophy in mice [12]. Nevertheless, the intrinsic proteins that inhibit PI3K activity and PHH have not yet been reported.

Pik3ip1 has recently been identified as a transmembrane protein that possesses a domain sharing homology with the PI3K regulatory subunit p85 [8]. This protein directly interacts with the catalytic subunit of PI3K, p110, and regulates the PI3K activity through its p85-like domain [8]. Pik3ip1 participates in the PI3K pathway, which is in many cellular functions such as T cell activation, carcinogenesis, and apoptosis [9,10,32]. Several studies have shown that silencing of Pik3ip1 increases PI3K activity in basal conditions [8,10]. In addition, liver-specific Pik3ip1 transgenic (TG) mice exhibit blunted PI3K activity [9]. However, the function of Pik3ip1 in the heart remains unknown.

**Fig 7:**
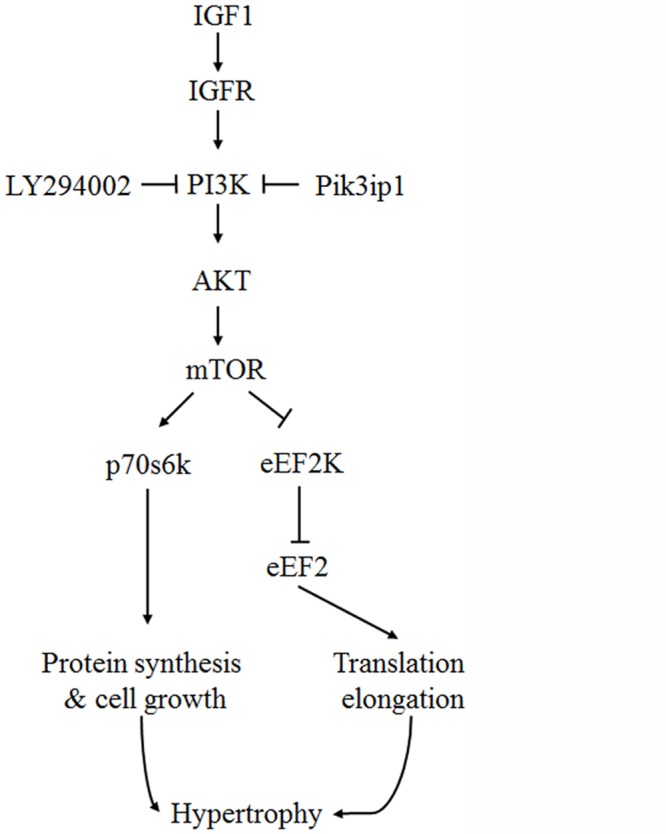
A working pathway model to show the effect of Pik3ip1 on exercise-induced cardiac hypertrophy. A schematic model of Pik3ip1-deficiency-mediated cardiomyocyte hypertrophy. Pik3ip1 inhibits PI3K activity through interacting with p110. Knockdown of Pik3ip1 could increase AKT activity in basal conditions. Activated AKT induces hypertrophy by activation of the mTOR pathway. mTOR increases cell growth and protein synthesis through activation of p70s6k and eEF2. Pik3ip1-deficiency-mediated cardiomyocyte hypertrophy is attenuated by treatment with the PI3K inhibitor, LY294002. In addition, Pik3ip1 can attenuate IGF1-induced hypertrophy by inhibiting PI3K activity.

In this study, we described the functional significance of Pi3kip1 in cardiomyocyte hypertrophy through its role in controlling the PI3K pathway. The main findings of this study are: (1) Pik3ip1 was predominantly expressed in cardiomyocytes, but not in fibroblasts, and it interacted with p110α (Fig 1), (2) Pik3ip1 knockdown increased myocardial growth and protein synthesis through the activation of the PI3K/AKT/mTOR pathway (Figs 2 and 3), (3) treatment with LY294002, a specific inhibitor of PI3K, confirmed that the Pik3ip1-deficiency-induced cardiomyocyte hypertrophy is due to PI3K pathway activation (Fig 4), (4) overexpression of Pik3ip1 negatively regulates PI3K pathway (Fig 5), and (5) Pik3ip1 expression was increased in the 4-weeks exercise-induced cardiac hypertrophy mouse model, which also showed an attenuation of AKT activity (Fig 6). Collectively, our study led to a novel finding that the PI3K binding partner, Pik3ip1, plays an important role in physiological cardiac hypertrophy through the PI3K pathway (Fig 7).

Cardiac-specific overexpression of AKT determines the heart and cardiomyocyte size by activating participating signaling proteins, suggesting that AKT is a hub signaling protein for the control of cardiac hypertrophy [33]. An important observation in the present study is that the regulation of AKT activation depends on Pi3kip1 expression level, indicating that Pi3kip1 level is essential for the modulation of cardiac hypertrophy. mTOR signaling has also been reported to enhance cardiac hypertrophy and to be deregulated often in cardiac diseases [34]. AKT activation can also enhance protein synthesis by activating mTOR signaling. Inhibition of mTOR activity by a specific inhibitor, rapamycin, has been shown to attenuate the development of cardiac hypertrophy [34]. mTOR can regulate protein synthesis through the modulation of p70s6k activity, which increases ribosomal biosynthesis and protein translation [22]. The present study showed that silencing of Pik3ip1 increased both mTOR and p70s6k activation, leading to protein synthesis in NRCMs.

AKT is known to be an important regulator of cardiac hypertrophy. Although AKT knockout mice showed normal heart function and phenotype, cardiac hypertrophy induced by exercise was attenuated in AKT knockout mice [35]. Moreover, transgenic mice expressing a constitutive active form of AKT initially showed PHH [33]. However, prolonged or excess expression of cardiac AKT caused PAH [36,37]. These studies suggested that the regulation of AKT activity could be an important factor in the control of the types of cardiac hypertrophy.

Considering the possible regulation of AKT activity in the 4-weeks exercised mice by Pik3ip1, PI3K negative regulator may play a role in blocking hyperactivation of PI3K. However, previous studies have shown that exercise training with treadmill or mild swimming program could continuously activate AKT in 4-weeks or even 8-weeks exercise training [38,39], suggesting that not only the type of exercise, but also the intensity of the exercise is important for the pattern of regulation of AKT activity by Pik3ip1.

Recent studies have reported that transcription factors such as Foxo3 and Cux1 are involved in the regulation of Pik3ip1 expression in brain tissue and cancer cells [40,41]. However, our preliminary studies using siRNAs for Foxo3 and Cux1 suggest that they may not be involved in the regulation of Pik3ip1 expression in the heart. Future studies are warranted to investigate the mechanism(s) underlying the exercise-training-induced Pik3ip1 upregulation and to identify the transcription factors involved.

## Supporting Information

S1 File Supplementary Figures and Tables. (DOCX)
