# Supplementary material for: Pik3ip1 Modulates Cardiac Hypertrophy by Inhibiting PI3K Pathway
Source: PLoS One. 2015 Mar 31;10(3):e0122251. doi: 10.1371/journal.pone.0122251 (PMC4380398; doi:10.1371/journal.pone.0122251)
Supplement: S1 File — (DOCX) [file pone.0122251.s001.docx]

**Supporting Information**

Supplemental Figure A. Isolation of cardiomyocytes and fibroblasts from neonatal rat hearts.

Supplemental Figure B. Pik3ip1 does not interact with p110γ

Supplemental Figure C. Silencing of Pik3ip1 does not lead to pathological cardiac hypertrophy.

Supplemental Figure D. Pik3ip1 does not attenuates AngII-induced cardiomyocyte hypertrophy.

Supplemental Figure E. Fetal genes were increased in TAC operated mice.

Supplemental Figure F. Pik3ip1 was increased in 4weeks exercised myocyte, not fibroblast.

Supplemental Table A. The primer sets for qRT-PCR and RT-PCR

Supplemental Table B. Echocardiographic analysis in TAC operated and exercised mice.


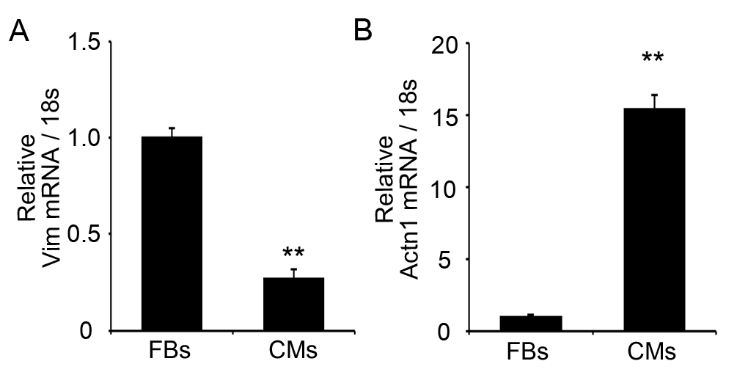


**Supplemental Figure A. Isolation of cardiomyocytes and fibroblasts from neonatal rat hearts.** (A, B) mRNA levels of *Vim* (A) and *Actn1* (B) were measured in cardiomyocytes (CMs) or fibroblasts (FBs) by qRT-PCR. (n = 3, ** p < 0.01 compared with fibroblasts, t test)

**
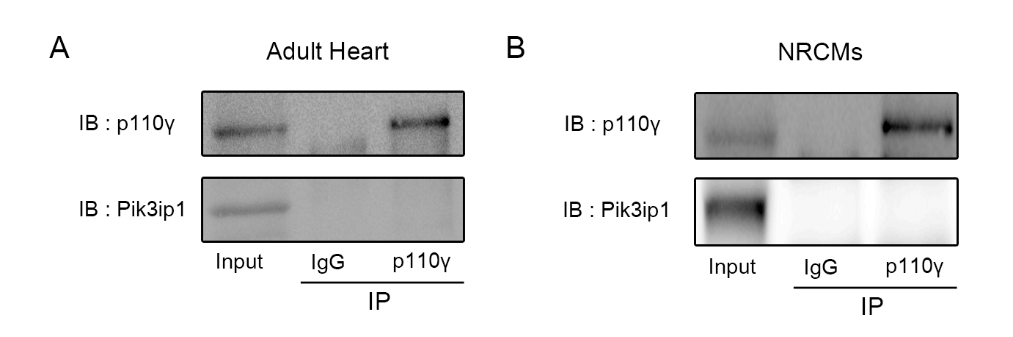
**

**Supplemental Figure B. Pik3ip1 does not interact with p110γ.** The interaction between Pik3ip1 and p110γ was analyzed in adult mouse heart tissue (A) and NRCMs (B) using anti-p110γ or anti-Pik3ip1 antibodies.


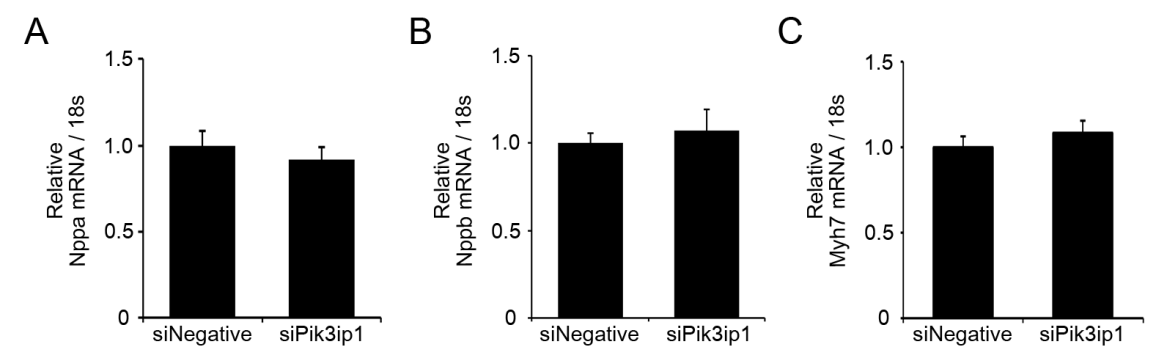


**Supplemental Figure C. Silencing of Pik3ip1 does not lead to pathological cardiac hypertrophy.** (A-C) qRT-PCR analysis of transcripts for *Nppa* (A), *Nppb* (B), and *Myh7* (C) in siNegative or siPik3ip1-transfected NRCMs. mRNA expression was normalized to 18S. (n = 3).

**

**

**Supplemental Figure D. Pik3ip1 does not attenuates AngII-induced cardiomyocyte hypertrophy.** NRCMs were infected with the indicated adenovirus for 24 h and subsequently treated with or without 100 nM AngII. (A) Extracts from adenovirus-infected NRCMs treated for 30 min with or without IGF1 were verified by the indicated antibodies. (B) Adenovirus-infected NRCMs were incubated for 24 h with or without AngII, after which protein synthesis was assessed using a leucine incorporation assay (n = 4, ** p < 0.01 compared with AdControl-infected NRCMs).

**

**

**Supplemental Figure E. Fetal genes were increased in TAC operated mice.** (A, B) qRT-PCR analysis of transcripts for *Nppa, Nppb, Myh7* in 2-weeks (A) and 4-weeks exercised mice (B) hearts. mRNA expression was normalized to 18S. (n = 3). (C, D) qRT-PCR analysis of transcripts for *Nppa, Nppb, Myh7,* in 1-week and 2-weeks TAC mice hearts. mRNA expression was normalized to 18S. (n = 3, ** p < 0.01 compared with Sham, t test).

**
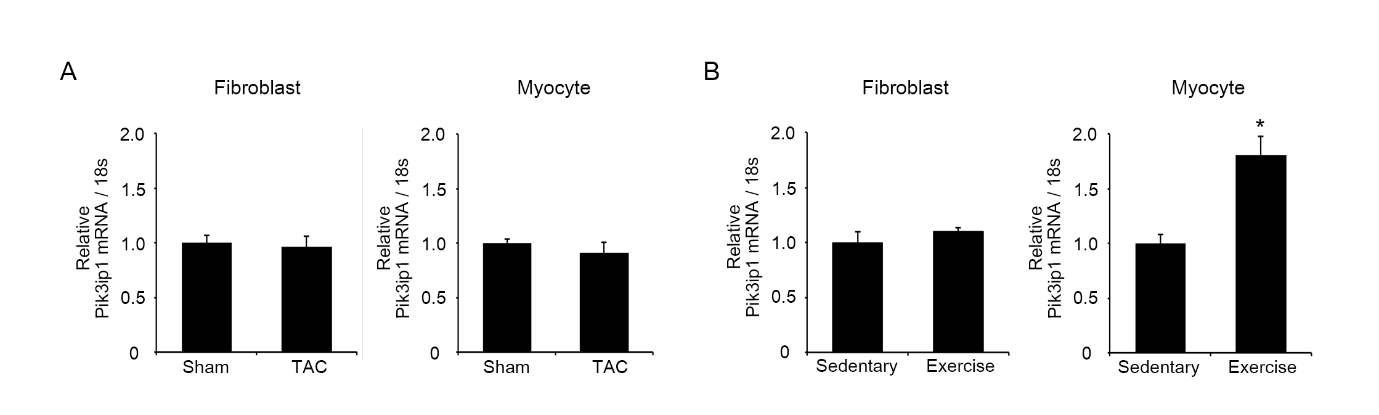
**

**Supplemental Figure F. Pik3ip1 was increased in 4-weeks exercised myocyte, not fibroblast.** (A, B) mRNA levels of Pik3ip1 were measured in 2-weeks TAC (A) and 4-weeks exercised mice (B) cardiomyocytes and fibroblasts using quantitative reverse transcription PCR (qRT-PCR). (n = 3, * p < 0.05, t test).

**Supplemental Table A. The primer sets for qRT-PCR and RT-PCR** F and R represent forward and reverse, respectively.

| Name of primer | Sequence |
| --- | --- |
| Mouse 18s-F | TTCTGGCCAACGGTCTAGACAAC |
| Mouse 18s-R | CCAGTGGTCTTGGTGTGCTGA |
| Mouse Pik3ip1-F | CCATGGAGCTGGAAGAGAAG |
| Mouse Pik3ip1-R | AGCTCCAATAGCGAGGATGA |
| Mouse Nppa-F | TCGTCTTGGCCTTTTGGCT |
| Mouse Nppa-R | TCCAGGTGGTCTAGCAGGTTCT |
| Mouse Nppb-F | AGGGAGAACACGGCATCATT |
| Mouse Nppb-R | GACAGCACCTTCAGGAGAT |
| Mouse Myh7-F | CGCATCAAGGAGCTCACC |
| Mouse Myh7-R | CTGCAGCCGCAGTAGGTT |
| Rat 18s-F | AAGTTTCAGCACATCCTGCGAGTA |
| Rat 18s-R | TTGGTGAGGTCAATGTCTGCTTTC |
| Rat Pik3ip1-F | TGGCAAATAAGGGCTTCCACA |
| Rat Pik3ip1-R | CCTGGATGGCAAAGGCAGA |
| Rat Nppa-F | ACCTGCTAGACCACCTGGAGGAG |
| Rat Nppa-R | CCTTGGCTGTTATCTTCGGTACCG |
| Rat Nppb-F | GCTGCTTTGGGCACAAGATAG |
| Rat Nppb-R | GGTCTTCCTACAACAACTTCA |
| Rat Myh7-F | AAGTCCTCCCTCAAGCTCCTAAGT |
| Rat Myh7-R | TTGCTTTGCCTTTGCCC |
| Rat Actn1-F | CGACATCAGGAAGGACCTGT |
| Rat Actn1-R | ACATCTGCTGGAAGGTGGAC |
| Rat Vim-F | ATGAAAGTGTGGCTGCCAAGAA |
| Rat Vim-R | GTGACTGCACCTGTCTCCGGTA |

**Supplemental Table B. Echocardiographic analysis in TAC operated and exercised mice.**

|  | 1week | | 2weeks | | 2weeks | | 4weeks | |
| --- | --- | --- | --- | --- | --- | --- | --- | --- |
|  | Sham | TAC | Sham | TAC | Sedentary | Swim | Sedentary | Swim |
| Number of animals | 5 | 5 | 5 | 6 | 5 | 6 | 5 | 5 |
| Heart weight/Body weight | 4.16 ± 0.04 | 5.62 ± 0.12 * | 4.24 ± 0.04 | 5.78 ± 0.17 * | 4.08 ± 0.13 | 4.20 ± 0.12 | 4.03 ± 0.05 | 4.77 ± 0.05 * |
| IVS, mm | 0.50 ± 0.04 | 0.76 ± 0.05* | 0.44 ± 0.03 | 0.72 ± 0.04* | 0.52 ± 0.04 | 0.52 ± 0.04 | 0.52 ± 0.02 | 0.58 ± 0.04 |
| LVPWd | 0.58 ± 0.04 | 0.74 ± 0.05* | 0.58 ± 0.06 | 0.71 ± 0.06* | 0.54 ± 0.06 | 0.54 ± 0.05 | 0.52 ±0.05 | 0.68 ± 0.04* |
| LVIDd | 0.40 ± 0.01 | 0.41 ±0.02 | 0.386 ± 0.03 | 0.42 ±0.02 | 0.40 ± 0.01 | 0.40 ± 0.01 | 0.39 ± 0.01 | 0.44 ± 0.01 |
| FS % | 41.8 ± 0.4 | 35.3 ± 1.6* | 42.0 ± 1.4 | 33.1 ± 0.4* | 36.5 ± 0.7 | 36.8 ± 0.6 | 37.3 ± 0.3 | 38.6 ± 1.1 |

| TAC indicates aortic transverse banding; all parameters indicated are LV diastolic values. LVID, LV internal diameter; IVS, interventricular septum; LVPW, LV posterior wall; FS, Fractional shortening. Values are mean ± SEM *P<0.05 vs Sham or Sedentary values |
| --- |
